# Influence of Tall Fescue *Epichloë* Endophytes on Rhizosphere Soil Microbiome

**DOI:** 10.3390/microorganisms9091843

**Published:** 2021-08-31

**Authors:** Kishan Mahmud, Kendall Lee, Nicholas S. Hill, Anaas Mergoum, Ali Missaoui

**Affiliations:** 1 Center for Applied Genetic Technologies, University of Georgia, Athens, GA 30602, USA; kishan.mahmud25@uga.edu; 2 Institute of Plant Breeding, Genetics and Genomics, University of Georgia, Athens, GA 30602, USA; kendall.lee94@uga.edu; 3 Department of Crop and Soil Sciences, University of Georgia, Athens, GA 30602, USA; nhill@uga.edu; 4 Department of Internal Medicine, School of Medicine and Health Sciences, University of North Dakota, Grand Forks, ND 58102, USA; anaas.mergoum@und.edu

**Keywords:** tall fescue, endophyte, soil, rhizosphere microbiome, plant-soil interaction

## Abstract

Tall fescue (*Lolium arundinaceum* (Schreb.) S.J. Darbyshire) often forms a symbiotic relationship with fungal endophytes (*Epichloë coenophiala*), which provides increased plant performance and greater tolerance to environmental stress compared to endophyte-free tall fescue. Whether this enhanced performance of tall fescue exclusively results from the grass–fungus symbiosis, or this symbiosis additionally results in the recruitment of soil microbes in the rhizosphere that in turn promote plant growth, remain a question. We investigated the soil bacterial and fungal community composition in iron-rich soil in the southeastern USA, and possible community shifts in soil microbial populations based on endophyte infection in tall fescue by analyzing the 16s rRNA gene and ITS specific region. Our data revealed that plant-available phosphorus (P) was significantly (*p* < 0.05) influenced by endophyte infection in tall fescue. While the prominent soil bacterial phyla were similar, a clear fungal community shift was observed between endophyte-infected (E+) and endophyte-free (E−) tall fescue soil at the phylum level. Moreover, compared to E− soil, E+ soil showed a greater fungal diversity at the genus level. Our results, thus, indicate a possible three-way interaction between tall fescue, fungal endophyte, and soil fungal communities resulting in improved tall fescue performance.

## 1. Introduction

Grasses cover almost 20% of the total land area on the planet [1] and are widely distributed ecosystems [2]. They offer important ecosystem services, such as providing forage for livestock [3], soil carbon sequestration [4], improved runoff quality [5], erosion control, climate regulation [6], and resistance to invasive species [7]. Many grass species are known to form symbiotic relationships with fungal endophytes [8] that led to eventual plant colonization of terrestrial environments [9]. Tall fescue (*Lolium arundinaceum* (Schreb.) S.J. Darbyshire), a cool-season perennial grass [10], is cultivated on an estimated 14-million hectares in the United States [11]. Tall fescue often forms an interdependent relationship with a shoot-specific fungal endophyte (*Epichloë coenophiala*) that produces ergot alkaloids that are toxic to livestock, causing fescue toxicosis or fescue foot [12,13]. To avoid *fescue toxicosis*, novel endophytes were identified and introduced into different tall fescue cultivars with non-toxic alkaloids such as lolines and peramines [14]. Although detrimental to livestock, tall fescue infected with *Epichloë coenophiala* has been shown to be persistent, exhibit better plant fitness, and offer improved ecosystem services over other grass species in pastures [15,16]. Endophyte infected tall fescue is a unique model to investigate the potential relationships between above and below-ground microbial communities. This potential relationship between the tall fescue, the endophyte, and the soil microbial communities might provide important insights to explore and clarify the plant’s resilience against environmental stress and climate change, different soil biogeochemical processes that influence soil health, and vital ecosystem services. It is well documented that soil microbial communities impart significant benefits in soil nutrient cycling, soil fertility status, and soil carbon sequestration that influences plant fitness and survival in varying terrestrial ecosystems [17,18,19]. 

The soil microbiome composition is at the forefront of evolutionary ecology where the primary focus is on the identification of the beneficial microbial communities and comprehending the extent of influence on plant performance and soil health [20,21,22]. Soil nutrient status plays a central role in impacting soil bacterial and fungal communities. This is particularly true for phosphorus (P), the least mobile macronutrient, found in the soil of the southeastern USA [23]. Due to its fixation with insoluble mineral-complex with iron (Fe) and aluminum (Al) oxides in acidic soil and with calcium (Ca) in alkaline soil, P is often limitedly released into the soil solution for root uptake [24,25]. Soil microbial communities, especially the phosphorus solubilizing microbial communities can secrete hydrolyzing enzymes, organic acids, protons, and phosphatases that can solubilize the organo-mineral complexes and release P and the associated mineral, eventually leading to the acquisition of unavailable P in soil by plants [26,27,28,29,30]. In return, the plant excreted rhizo-deposits and root architecture contribute significantly in the rhizosphere microbial communities [31]. Endophyte infection in tall fescue may offer a competitive advantage to non-infected fescue by influencing the soil microbial processes and soil microbial communities [32,33,34]. Additionally, the quantity and type of root exudates and rhizo-deposits change with different stages of plant development [35,36,37], thus, creating a resource partitioning in the soil that subsequently leads to niche partitioning [38,39,40]. In turn, given the rhizosphere origin of endophytic microbial populations, soil bacterial and fungal community composition may regulate the plant endophytic diversity and community composition [41]. In earlier studies based on the endophyte infection in tall fescue, shifts in soil microbial (bacterial and fungal) community structure and soil food webs have been reported [42,43]. These plant–fungal associations, especially in grass species, define a significant two-faceted interaction: (i) the collaboration gradient (above-ground) [44]; and (ii) root exudates mediated influence (below-ground) [45]. The first interaction describes how the plant–fungal symbiosis impacts nutrient foraging; promotes plant growth [46]; provides resilience against biotic stress, such as plant pathogens [47]; and abiotic stress, such as drought and salt tolerance [48]. The second interaction highlights the fungal communities associated with the rhizosphere communities, facilitates soil nutrient cycling and nutrient acquisition [49], organic matter decomposition [50,51], synthesis of phytohormones for root utilization [52], resistance against nematodes [53], and protection against pathogens [54]. Thus, determining the endophyte-facilitated soil microbial processes and the subsequent soil microbial response contributing to increased plant production and stress tolerance may carry significant economic and ecological importance for sustainable agricultural practices [55]. Our objective was to explore the diversity of the soil bacterial and fungal communities associated with tall fescue rhizosphere and investigate whether the bacterial and fungal populations differ based on the presence of endophyte in tall fescue. 

## 2. Materials and Methods

### 2.1. Site Description

The study site was in the southeastern region of the USA at the J. Phil Campbell (JPC) Research and Education Center (33°52′ N, 83°27′ W) and Iron Horse Farm (IHF) (33°72′ N, 83°30′ W) in Watkinsville, Georgia. The soil at JPC is a fine kaolinitic, thermic Typic Kanhapludults in the Cecil sandy loam series with a 2% to 6% slope. The soil at IHF is Pacolet sandy clay loam, with a 6% to 10% slope [56]. The region has 123 cm average annual rainfall and an average minimum and maximum temperature of 10.4 °C and 22.5 °C, respectively. Soil sampling for this study was completed in October 2019, following a summer that according to the National Oceanic and Atmospheric Administration had the hottest July on record, since the late 1800s. The research plots were established in the fall of 2014 with 750 different tall fescue accessions. Each tall fescue accession was planted in 1.5 m single row: 0.75 m space between plots within the range and 1.5 m between ranges. Since its establishment, the plots were fertilized with inorganic fertilizers (N-P-K) in October 2014 and regular clippings of the grass were performed every spring. 

Table 1 shows the average atmospheric temperature, soil temperature, and average rainfall between 2014 to 2019. 

### 2.2. Soil Sampling and Tall Fescue Plants

We sampled soil from 48 different tall fescue accessions’ rhizospheres with a hand soil probe (2.5 cm diameter) to a depth of 0–15 cm. All soil samples were kept refrigerated at 4 °C. The soils were then air-dried, ground, and passed through a 2 mm sieve for soil nutrient analysis. Out of 48 tall fescue ranges, 43 ranges were planted at JPC and five were planted at the IHF site. We selected nine tall fescue cultivars with no endophytes (E−) and 35 cultivars with endophyte infection (E+), among which 21 were infected with novel-endophytes and 14 with wild-type, toxic endophytes. At the time of soil sampling, for the purpose of microbial analysis, soil samples were immediately separated and kept at −20 °C until the soil genomic DNA was extracted (see below Section 2.3). 

### 2.3. DNA Extraction, PCR Amplification, and 16S rRNA Gene and ITS Gene Sequencing

DNA Extraction, PCR Amplification, and 16S rRNA Gene and ITS Gene Sequencing from homogenized and frozen soil (0.25 g), soil DNA was extracted using QIAGEN DNeasy PowerSoil Kit (DNeasy PowerSoil Kit Handbook, May 2017, Qiagen, Valencia, CA, USA). Soil DNA quality and concentration were assessed by a NanoDrop 2000 spectrophotometer (Thermo Scientific, Waltham, MA, USA). Extracts were stored at −20 °C until further analysis. A bacterial sequencing library targeting the bacterial 16S rRNA genes was prepared using primer sets from PacBio 16S protocol (V1-V9 regions) [57]; 27F27F (AGRGTTYGATYMTGGCTCAG)/14292R (RGYTACCTTGTTACGACTT). For the fungal sequencing library, we targeted the ITS region and used ITS1-F Forward (CTTGGTCATTTAGAGGAAGTAA)/ITS2-R Reverse (GCTGCGTTCTTCATCGATGC) to amplify the ITS region. The sequencing workflow was as follows: (i) Multiplexing with PacBio Barcoded Universal Primers; (ii) AMPure PB bead purification; (iii) Pooling Barcoded Amplicons; (iv) SMRTbell Library Construction; (v) Purification of SMRTbell Templates; (vi) Anneal and Bind SMRTbell Templates; and (vii) Sequencing on PacBio Sequel II System. The first-round amplification PCR conditions were 95 °C for 180 s, followed by 20 cycles of 95 °C for 30 s, 57 °C for 30 s, and 72 °C for 60 s with universal primer-tailed 16S primers and ITS1 primers. The second-round amplification PCR conditions were 95 °C for 30 s, 57 °C for 30 s, and 72 °C for 60 s for 20 cycles with PacBio Barcoded Universal Primers. SMRTbell libraries were prepared by using PacBio Barcoded Universal Primers for Multiplex SMRT Sequencing. Then, PacBio’s single-molecule circular consensus sequencing (CCS) reads were generated for full-length 16S rRNA genes and ITS gene (accuracy of 99%). The CCS reads were de-multiplexed using the software “lima” in SMRT Analysis software version 2.3.0. To generate bam files followed by a conversion to Fastq files via bam2fastq. 

### 2.4. Data Analysis

The CCS reads were processed with DADA2 software packages (16S rRNA gene and ITS specific workflow) (version 1.8) [58], and analyzed with phyloseq for alpha and beta diversity (version 1.25.2) [59]. For 16s rRNA gene CCS data, the DADA2 workflow follows primer trimming, quality filtering, and de-replication. Amplicon sequence variants (ASVs) were inferred after learning the error rates. Afterward, the “removeBimeraDenovo” command was used to remove chimeras. Finally, we used the SILVA nr v132 train set to assign taxonomy. For the fungal data analysis, we followed the ITS-specific variation of the DADA2 package. In the fungal DADA2 workflow, after orienting the primers, we used a specialized primer/adapter removal tool “cutadapt” [60]. After primer removal, the next steps consist of quality filtering, de-replication, inferring ASVs after error learning, and finally removing chimeras. We used UNITE ITS database for taxonomic assignments [61]. The ASV tables from DADA2 pipelines were imported into phyloseq to make phyloseq objects and to calculate alpha and beta diversity. Sigma Plot 11 was used to generate figures depicting percentage of bacterial and fungal populations in soil. 

### 2.5. Statistical Analysis

Analysis of variance with JMP PRO 15 software (JMP^®^, Version 15. SAS Institute Inc., Cary, NC, USA, 1989–2019) was used to determine differences in soil pH, inorganic nitrogen, nitrate content, calcium, potassium, magnesium, phosphorus, and zinc, between endophyte-free fescue soil, non-toxic endophyte-infected fescue soil, and toxic endophyte-infected fescue soil samples (*p* < 0.05). Comparisons between multiple means of different soil nutrient content were completed with Tukey’s HSD (*p* < 0.05). 

## 3. Results

### 3.1. Soil Chemical Properties

There were no significant differences in soil pH and soil nutrient content between E− and E+ tall fescue soil, except for plant-available phosphorus in soil (Table 2). The E+ tall fescue soil had higher plant-available P compared to the E− tall fescue soil. Between endophyte-free, non-toxic, and toxic endophyte-infected tall fescue soil, non-toxic endophyte-infected soil had significantly greater plant-available P compared to the rest (Table 2). Although, Zn content in soil was not statistically significant between the E− and E+ tall fescue soil, three endophyte-infected tall fescue soil samples, accession 1062, 1064, and Bar Optima had higher soil Zn content.

### 3.2. Soil Bacterial Abundance, Diversity, and Community Composition 

**Figure 1:**
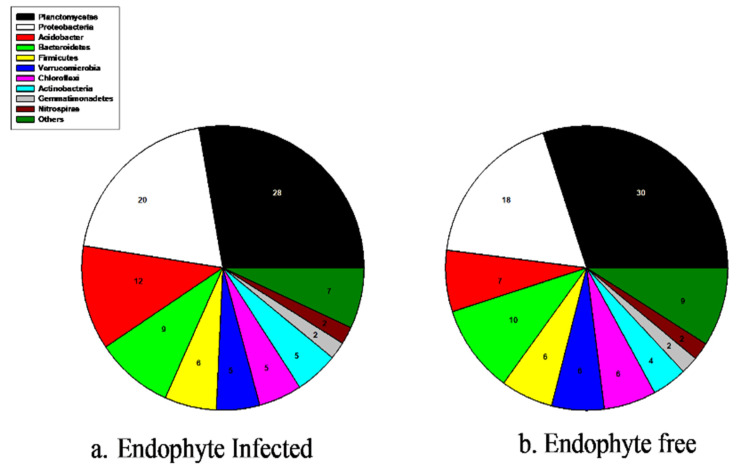
(**a**,**b**) Prominent bacterial phyla in soil based on endophyte presence in tall fescue.

We identified 1212 and 3411 bacterial amplicon sequence variants (ASVs) in the E− and E+ tall fescue soil collected from the tall fescue plots, respectively. We identified 18 phyla, 29 classes, 72 orders, 111 families, and 151 bacterial genera in E+ tall fescue soil. In E− tall fescue soil, we identified 14 phyla, 29 classes, 45 orders, 88 families, and 97 bacterial genera. The Shannon diversity index (SDI) highlights the species’ richness and evenness among the entire community; the higher number indicates higher diversity. The mean bacterial Shannon diversity index was lower overall and not statistically significant between the E− (mean H′ = 4.5) and E+ (mean H′ = 4.0) soil. Additionally, bacterial beta-diversity presented with principal coordinate analysis (PCoA) based on Bray–Curtis dissimilarities showed no significant differences between soil microbial communities based on the presence of endophyte in tall fescue. The prominent bacterial phylum in both E− and E+ tall fescue soil was Planctomycetes (Figure 1a,b). In E+ tall fescue soil, the abundance of phyla from greatest to lowest was as follows: Planctomycetes (28%) > Proteobacteria (20%) > Acidobacteria (12%) > Bacteroidetes (9%) > Firmicutes (6%) > Verrucomicrobia, Chloroflexi and Actinobacteria (5%) > Gemmatimonadetes and Nitrospira (2%) (Figure 1a). For E− tall fescue soil, from greatest to lowest abundance of the prominent bacterial phyla was as follows: Planctomycetes (30%) > Proteobacteria (18%) > Acidobacteria (7%) > Bacteroidetes (10%) > Firmicutes, Verrucomicrobia, Chloroflexi (6%) > Actinobacteria (4%) > Gemmatimonadetes and Nitrospira (2%) (Figure 1b).

**Figure 2:**
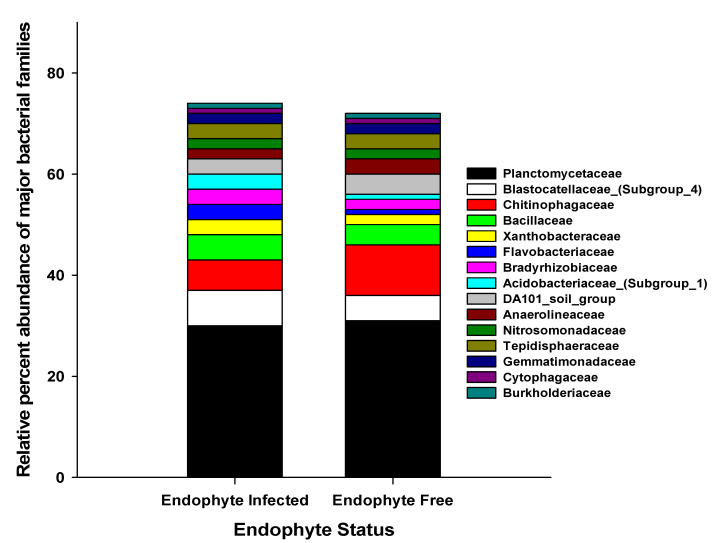
Relative percent abundance of bacterial families in tall fescue soil.

Prominent bacterial families between E− and E+ soil was *Planctomycetaceae*, *Balstocatellaceae_(subgroup_4), Chitinophagaceae,* and *Bacillaceae* (Figure 2). Moreover, we found several characteristics including: nitrogen-utilizing, phosphorus solubilizing, bio-controller, chitin degrading, nitrate reducers, drought and salt tolerant, and other nutrient solubilizing bacterial families, for instance, *Planctomycetaceae*, *Xanthobacteraceae*, *Flavobacteriaceae*, *Bradyrhizobiaceae*, *Acidobacteriaceae_(Subgroup_1)*, *DA101_soil_group*, *Anaerolineaceae*, *Nitrosomonadaceae*, *Tepidisphaeraceae*, *Gemmatimonadaceae*, *Cytophagaceae*, *Burkholderiaceae*, and *Comamonadaceae* (Figure 2 and Supplemental Material S1). Endophyte-free tall fescue soil had higher *Planctomycetaceae* (31% of Planctomycetes) and *Chitinophagaceae* (10% of Bacteroidetes) compared to the E+ soil, where the *Balstocatellaceae_(subgroup_4)* (7% of Acidobacteria) and *Bacillaceae* (5% of Firmicutes) were higher (Figure 2). 

### 3.3. Soil Fungal Abundance, Diversity, and Community Composition 

**Figure 3:**
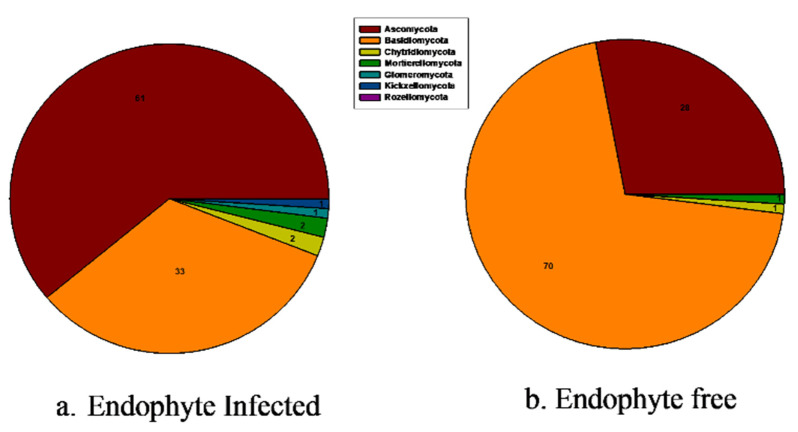
(**a**,**b**) Distribution of major fungal phyla in soil based on endophyte presence in tall fescue.

**Figure 4:**
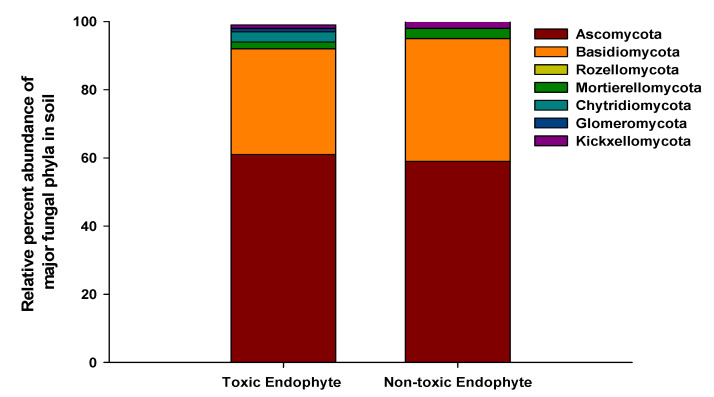
Prominent fungal phyla in soil based on endophyte toxicity in tall fescue.

**Figure 5:**
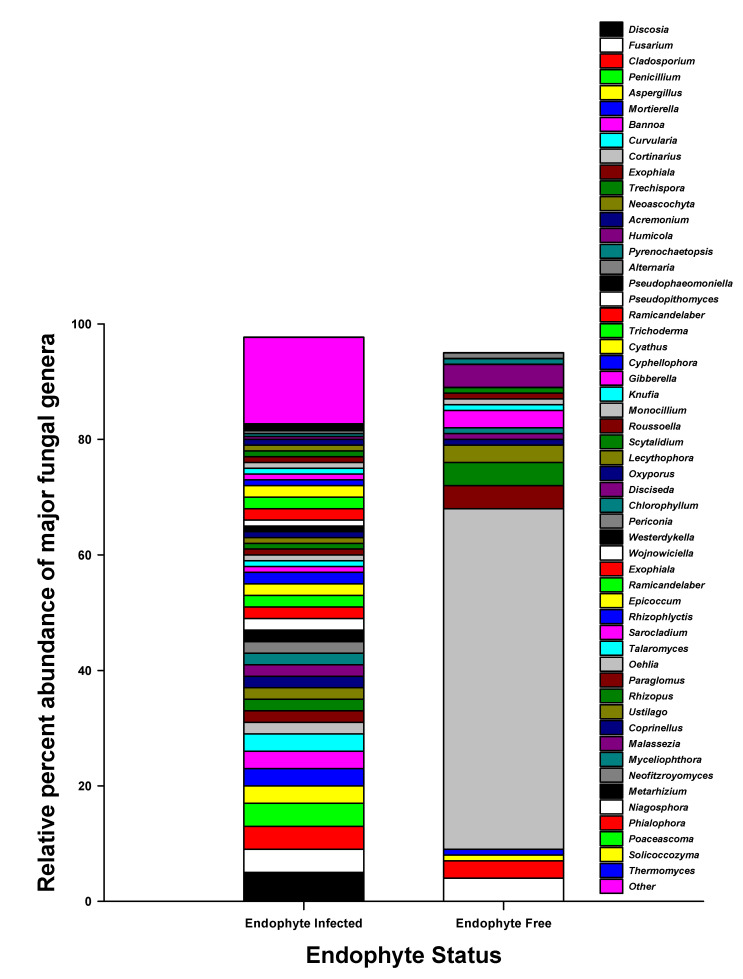
Relative percent abundance of fungal genres in tall fescue soil.

We identified 71 and 652 fungal ASVs in the E− and E+ tall fescue soil collected from the tall fescue plots, respectively. In E+ tall fescue soil, we identified 6 phyla, 24 classes, 43 orders, 76 families, and 112 bacterial genera. We identified 3 phyla, 6 classes, 10 orders, 18 families, and only 19 bacterial genera in E− tall fescue soil. In both E− and E+ tall fescue soil, the dominant fungal phyla consisted of Basidiomycota and Ascomycota, respectively (Figure 3a,b). The mean fungal Shannon diversity index was lower overall and was not statistically significant between the E− (mean H′ = 1.21) and E+ (mean H′ = 1.27) soil. Fungal beta diversity presented with principal coordinate analysis (PCoA) based on Bray–Curtis dissimilarities also showed no significant differences. Interestingly, however, we did observe a fungal community shift between E− and E+ tall fescue soil. While Basidiomycota (70%) dominated E− soil, E+ soil had Ascomycota as the prominent phylum (Figure 3a,b). Based on the toxicity status of the endophyte presence in the tall fescue, E+ soil showed a similar percent abundance at phyla level where both toxic and non-toxic infected tall fescue soil had Ascomycota as the prominent phylum (Figure 4). Arbuscular mycorrhizal fungi (AMF) belonging to Glomeromycota phylum (1% of the total fungal abundance) were identified in only E+ fescue soil (Figure 3a). In the case of fungal genera, while E+ soil had no such genus that exceeded more than 5% of the total abundance, the most prominent genus in E− soil belonged to *Cortinarius* (59% of Basidiomycota) (Figure 5). Interestingly, we measured greater diversity at the genus level in E+ soil (111 genera) compared to E− soil (19 genera). These different fungal genera have been shown to contribute to: plant growth promotion, plant-pathogen suppression, lignin degradation, nitrogen utilization, phosphorus solubilization, biodegradation, phytohormone production, and provides resistance against abiotic stresses such as drought, salt intrusion, and cold tolerance, etc. (Figure 5 and Supplemental Material S2).

## 4. Discussion

Despite the intricate nature of soil microbial populations, we found common patterns in bacterial community responses in the soil to the endophyte presence in tall fescue and our results from soil bacterial analysis indicate that the endophyte presence in tall fescue might have had a subtle effect on the bacterial community composition. Contrasting results, however, have been reported on the impact of the endophyte presence in grass species on soil microbial community composition and microbial functions [62]. For instance, soil microbial communities may alter microbial functions due to above-ground endophyte infection of grass species, such as microbial carbon and nitrogen mineralization [32,63,64,65]. Furthermore, endophyte infection of above-ground plant material stimulated below-ground microbial functions primarily due to endophyte-induced rhizodeposition [66]. In our study, the lack of bacterial diversity in community composition perhaps can be speculated to the soil micro-niche effect [67]. Due to the size of bacteria, they are expected to be in direct contact with their immediate surroundings, but often these micro-niches have a different composition from the soil matrix [68]; thus, plant roots may never come into direct contact with the bacterial communities living in these niches and perhaps never influence the community composition of the bacteria living in soils [69]. 

In our study, Planctomycetes were the dominant oligotrophic phylum (r-strategists) found in both E+ and E− tall fescue rhizosphere soils and they are well suited for nutrient-poor soil indicated by lower soil carbon and phosphorus [70,71,72,73]. They are thought to be crucial in soil organic carbon and complex carbon turnover, nitrogen cycling, and subsequently for soil nutrient availability [74,75,76]. The second dominant bacterial phylum for both E+ and E− tall fescue soil was a versatile group of copiotroph, known as Proteobacteria, that responds to readily available carbon in soil [75,77]. Additionally, these Proteobacteria follow a fast growth pattern in the soil, which consequently may act as a plant growth promoter by releasing soil macro and micro-nutrients from organo-mineral complexes [78,79], especially under copiotroph environments [80]. It is well documented that by producing metabolites of fungal-origin, E+ tall fescue has a competitive advantage over E− grasses, particularly against climatic and edaphic stress, protection against herbivores, enhanced nutrient acquisition, for instance soluble P in nutrient-poor soils [81,82,83]. In our study, another oligotroph microbial taxa, Acidobacter, was found in greater relative percent abundance in E+ tall fescue rhizosphere soil, like Planctomycetes, offers efficient carbon and nitrogen cycling from soil organic matter that can consequently be used as a readily available nutrient source for the E+ plants [75,84]. The Proteobacteria to Acidobacteria (P/A) ratio may serve as a general indicator of soil nutrient status; a low P/A ratio indicates oligotrophic soil environment and a high P/A ratio suggests nutrient richness [85]. In our study, the percent abundance ratio of Proteobacteria/Acidobacteria (P/A) was lower in E+ tall fescue rhizosphere soil (1.66) compared to E− tall fescue rhizosphere soil (2.57). In general, E− tall fescue performs poorer in overall plant fitness and persistence [86], despite the higher soil nutrient status (indicated by high P/A), compared to E+ infected fescue, possibly due to the lower percent abundance of the Acidobacter phylum. Additionally, known copiotrophs, such as Bacteroidetes and Verrucomicrobia, were also present in relatively lower abundance, possibly, due to the overall lower nutrient concentration of the study site [87,88]. 

In the case of fungal community composition in soil, endophyte presence in tall fescue showed a clear shift in fungal phyla in the rhizosphere. In agroecosystems, strong evidence of multilateral interactions between plant population, soil fungi, and soil solution composition has been discovered [69,89,90,91]. The complex fungal community structure and greater diversity enable enhanced organic matter decomposition, thereby promoting higher nutrient absorption by plants and accelerated soil nutrient cycling [92,93,94]. The plants act as the energy source for the soil fungal population by releasing photosynthetic carbon and secondary metabolites in soil [95,96,97,98,99], thus creating a feedback loop. Thus, soil fungal diversity has a remarkable influence on the fitness of the plant population, soil nutrient composition, and is vastly influenced by the presence of endophytes in plants [100,101,102]. The three prominent fungal phyla in soil are the Ascomycota, Zygomycota, and Basidiomycota [89], and our study site was dominated by either Ascomycota or Basidiomycota depending on the presence of endophytes in tall fescue (not the type of endophytes; toxic or non-toxic). The lower SDI measured for the fungal population in the soil, both E+ and E−, may have been due to the overall higher soil pH of the study site; fungi generally grow better in acidic conditions [103], whereas, our study site had an average soil pH of 6.5. The greater relative abundance of Ascomycota and Basidiomycota in E+ and E− tall fescue rhizosphere soil, respectively, suggests that the presence of endophyte in tall fescue affects the rhizosphere fungal community structure, possibly through a combination of: (i) alkaloids such as loline or peramine excretion in the host grass [104,105]; (ii) production of VOCs and other biochemical induced by the tall fescue [106,107]; and finally, (iii) higher rhizodeposition [108], all of which finally contribute to increased resource availability for soil fungi. This, therefore, is an indication of a three-way relationship between the plant (tall fescue), fescue-dwelling fungal endophytes, and the soil fungal communities [109]. Furthermore, significantly greater plant-available P in E+ soil compared to E− soil (Table 2), particularly in non-toxic E+ soil, suggests the unique contribution of a less studied novel endophyte-host associations to plant nutrition under limited soil plant-available P [110]. This plant-available P in conjunction with the combined presence of Ascomycota, Basidiomycota, and Glomeromycota in E+ tall fescue soil in the rhizosphere is likely to contribute to plant and soil microbial communities’ growth [111]. A highly diverse soil microbial community can withstand the changing environment, show greater resilience, and may bring stability in ecosystem functioning [112,113,114,115]. The observed higher diversity of fungal genera in E+ tall fescue soil is particularly important under a stressed environment because of their impact on plant growth and higher stress amelioration [20,116]. Often, carbon acquisition can be strictly limited under abiotic stress, such as drought, and the plant-associated microbial communities lacks the necessary resources to sustain [117]. However, soil fungal communities may indirectly stimulate photosynthesis in plants by providing necessary nutrients [118]. Thus, the presence of a complex fungal assemblage at genus level in E+ tall fescue soil suggests (Figure 5) that root excreted rhizo-deposits from E+ tall fescue into the soil may have enhanced the mobilization or recruitment of beneficial rhizosphere fungal communities, and in turn, these different soil fungal communities possibly could provide greater fitness and resilience to the plant [119,120,121]. In addition, a greater number of fungal genera in the soil is also important in offering higher functional redundancy for both “basic” and “rare” soil functions [121], particularly under disturbed environments, hence, the greater distribution of different functional groups is a clear indicator of greater functional redundancy [78] in E+ soil compared to E− soil. 

## 5. Conclusions

Our study suggests that a three-way mutualistic relationship exists between tall fescue, fungal endophyte, and the soil rhizosphere communities, particularly the soil fungal community. This study reveals that while there was a subtle change in the soil bacterial population based on endophyte presence in above-ground tall fescue, prominent changes were observed in the fungal community at the genus level compared to the endophyte-free soil. These results point to the possibility that the different soil nutrient acquisition and environmental stress tolerance imparted by endophytes on tall fescue is probably the result of mobilization or recruiting of beneficial rhizosphere microorganisms; however, further field trials of different endophytes in common plant genetic backgrounds are needed to confirm this.

## Figures and Tables

**Table 1:** Average maximum, minimum, and daily atmospheric temperature (°C), average soil temperature (0–15 cm) (°C), and average rainfall (mm) during summer months (June, July, and August) from 2015 to 2019.

Season	Maximum Atmospheric Temperature (°C)	Minimum Atmospheric Temperature (°C)	Daily Atmospheric Temperature (°C)	Daily Soil Temperature (°C) at 15 cm Depth	Average Rainfall (mm)
Summer 2015	32	21	26	28	391
Summer 2016	33	21	27	30	295
Summer 2017	30	20	25	28	517
Summer 2018	31	20	25	28	392
Summer 2019	31	20	25	29	304

**Table 2:** Mean soil nitrogen (N), calcium (Ca), potassium (K), magnesium (Mg), manganese (Mn), phosphorus (P), and zinc (Zn) (mg/kg) content.

	pH	NH_4_^+^- N	NO_3^−^_ N	Ca	K	Mg	Mn	P	Zn
Endophyte-Free Soil	6.53 ^a^	3 ^a^	244 ^a^	801 ^a^	40 ^a^	105 ^a^	17 ^a^	26 ^b^	1.0 ^a^
Endophyte-Infected Soil (Toxic)	6.59 ^a^	2 ^a^	270 ^a^	681 ^a^	43 ^a^	93 ^a^	17 ^a^	38 ^a^	1.17 ^a^
Endophyte-Infected Soil (Non-toxic)	6.59 ^a^	3 ^a^	245 ^a^	731 ^a^	45 ^a^	99 ^a^	16 ^a^	33 ^ab^	1.07 ^a^

Different lower-case letters indicate a significant difference between endophyte-free soil, toxic endophyte-infected soil, and non-toxic endophyte-infected soil (*p* < 0.05).

## Data Availability

The datasets during and/or analyzed during the current study is available from the corresponding author on reasonable request.

## Supplementary Materials

The following are available online at https://www.mdpi.com/article/10.3390/microorganisms9091843/s1, Supplemental Material S1: Prominent bacterial families (%) present in endophyte infected and endophyte free tall fescue soil, Supplemental Material S2: Prominent fungal genres (%) present in endophyte infected and endophyte free tall fescue soil.
