# Supplementary material for: Influence of Tall Fescue Epichloë Endophytes on Rhizosphere Soil Microbiome
Source: Microorganisms. 2021 Aug 31;9(9):1843. doi: 10.3390/microorganisms9091843 (PMC8468716; doi:10.3390/microorganisms9091843)
Supplement: Supplementary file 1 [file microorganisms-09-01843-s001.zip › Supplemental Material S1.pdf]

Supplemental Material S1- Prominent bacterial families (%) present in endophyte infected and endophyte free tall fescue soil

Phylum	Order (EI, EF)	Family	Endophyte Infected (EI)	Endophyte Free (EF)
Proteobacteria	Rhizobiales (38, 37)	*Bradyrhizobiaceae* (N-fix) (Myc-Symb)	26	26
		Hyphomicrobiaceae (Pht)	13	18
		Rhizobiales_Incertae_Sedis (N-Fix)	10	10
		Xanthobacteraceae (N-Fix)	35	30
		Rhodobiaceae (N-Fix)	4	6
		Phyllobacteriaceae	4	Abs
		Methylobacteriaceae (PGP)	3	Abs
	Rickettsiales (13, 12)	*Mitochondria*	86	77
	Xanthomonadales (6, 14)	*Xanthomonadales_Incertae_Sedis*	80	75
		Xanthomonadaceae (PP)	5	9
	Rhodospirillales (7, 6)	*Rhodospirillales_Incertae_Sedis*	20	33
		Rhodospirillaceae (N-fix)	14	22
		Acetobacteraceae	26	11
	Nitrosomonadales (8, 9)	*Nitrosomonadaceae* (AOB)	100	100
	Burkholderiales (7, 3)	*Comamonadaceae* (Dntf) (Myc-Symb)	38	54
		Burkholderiaceae (PSB) (ZnSb) (DrtTol)	38	23
		Oxalobacteraceae (Myc-Symb)	24	15
	Sphingomonadales (3, 25)	*Sphingomonadaceae* (Biocon) (PhtRem)	96	100
	Desulfuromonadales (1, 5)	*Geobacteraceae* (FeRd) (SlfRB)	100	100
	Desulfurellales (5, 4)	*Desulfurellaceae* (SlfRB)	100	100
Planctomycetes	Planctomycetales (88 ,100)	*Planctomycetaceae* (NH3-Ox)	100	95
		Phycisphaeraceae	18	5
Verrucomicrobia	Chthoniobacterales (99,98)	*DA101_soil_group*	86	86
		Xiphinematobacteraceae	3	7
		Chthoniobacteraceae	7	7
	Verrucomicrobiales (6, 2)	*Verrucomicrobiaceae*	100	100

Bacteroidetes	Sphingobacteriales (68, 87)	*Sphingobacteriaceae (Biocon)*	10	10
		Chitinophagaceae	84	89
	Cytophagales (7, 8)	*Cytophagaceae*	100	100
	Flavobacteriales (25, 4)	*Flavobacteriaceae (PGPR)*	98	80
		NS9_marine_group	2	20
Nitrospirae	Nitrospirales (100, 100)	*Nitrospiraceae (NB)*	69	70
		0319-6A21(NB)	31	30
Acidobacteria	Blastocatellales (67,79)	*Blastocatellaceae_(Subgroup_4)*	100	100
	Acidobacteriales (25,0)	*Acidobacteriaceae_(Subgroup_1) (NitR)*	100	Abs
	Solibacterales (1,0)	*Solibacteraceae_(Subgroup_3)*	100	Abs
Gemmatimonadetes	Gemmatimonadales (100, 100)	*Gemmatimonadaceae*	100	100
Actinobacteria	Solirubrobacterales (27, 43)	*Elev-16S-1332*	81	61
		0319-6M6	11	11
		Solirubrobacteraceae	Abs	11
	Micrococcales (17, 21)	*Micrococcaceae (SlntRlf)*	91	100
	Gaiellales (20, 10)	*Gaiellaceae*	20	25
	Acidobacteriales (0, 7)	*Acidobacteriaceae_(Subgroup_1)*	Abs	100
	Corynebacteriales (10, 0)	*Mycobacteriaceae*	100	Abs
	Acidimicrobiales (14, 0)	*Acidimicrobiaceae*	5	Abs
Chloroflexi	Anaerolineales (61 , 53)	*Anaerolineaceae*	100	100
	Ktedonobacterales (23, 23)	*HSB_OF53-F07*	22	39
		Ktedonobacteraceae	48	16
		Thermosporotrichaceae	5	Abs
	Chloroflexales (5, 5)	*Roseiflexaceae*	100	100
Firmicutes	Bacillales (84, 65)	*Bacillaceae (PSB)*	80	84
		Paenibacillaceae (PSB)	8	10
		Alicyclobacillaceae	4	6
	Clostridiales (6, 13)	*Clostridiaceae_1(PP)*	36	40
		Gracilibacteraceae		10
		Peptostreptococcaceae	Abs	30
		Ruminococcaceae	Abs	10
		Lachnospiraceae	64	10
	Erysipelotrichales (10, 22)	*Erysipelotrichaceae*	100	100

Abbreviations for bacterial functions within EI and EF rhizosphere soil: AnPhtB, anaerobic photosynthetic bacteria; AOB, Ammonia oxidizing bacteria; BC, bio-controller; DnB, denitrifying bacteria; FBC, fungal bio-controller; FeRB, iron reducing bacteria; N-fix, nitrogen fixing bacteria; NOA, nitrite oxidizing Archaea; NOB, nitrite oxidizing bacteria; NitR, nitrate reducers; ; Pdtx, Phosphate detoxifier; PGP, plant growth promoter; Pht, phototrophic Bacteria; PP, Plant Pathogen; PrSlfB, purple sulfur reducing bacteria; PSB, phosphorus solubilizing bacteria; SlfRB, sulfur reducing bacteria and UD, undetermined. *Abs = Absent. Percent abundance of order is given in parenthesis for respective families.
