# Supplementary material for: Influence of Tall Fescue Epichloë Endophytes on Rhizosphere Soil Microbiome
Source: Microorganisms. 2021 Aug 31;9(9):1843. doi: 10.3390/microorganisms9091843 (PMC8468716; doi:10.3390/microorganisms9091843)
Supplement: Supplementary file 1 [file microorganisms-09-01843-s001.zip › Supplemental Material S2.pdf]

Supplemental Material S2- Prominent fungal genres (%) present in endophyte infected and endophyte free tall fescue soil

**Phylum**	**Family** (EI, EF)	**Genus**	**Endophyte Infected (EI)**	**Endophyte Free (EF)**
Ascomycota	Aspergillaceae (8, 5)	*Aspergillus* (PSF)	41	100
		Penicillium (PSF)	59	Abs
	Chaetomiaceae (5, 5)	*Humicola* (BioRm)	40	100
		Myceliophthora (LigCDgrd)	11	Abs
		Corynascella (Decomp) (Sidphr)	5	Abs
		Chaetomium (Decomp) (Sidphr)	5	Abs
		Acrophialophora	22	Abs
	Cladosporiaceae (5, 10)	*Cladosporium* (BioRm)	100	100
	Cucurbitariaceae (3, 5)	*Pyrenochaetopsis* (N-Ut)	100	100
	Didymellaceae (4, 10)	*Neoascochyta* (PP)	40	100
		Epicoccum (Biocon) (PP)	40	Abs
		Didymella (Decomp)	13	Abs
		Stagonosporopsis (PP) (MnOx)	7	Abs
	Helotiaceae (1, 5)	*Scytalidium* (SecMtbl)	50	100
		Articulospora	50	Abs
	Herpotrichiellaceae (4, 14)	*Exophiala* (BioRm)	54	100
		Phialophora (BioRm)	8	Abs
	Hypocreaceae (3, 5)	*Monocillium* (Biodg)	30	100
		Trichoderma (Decomp) (Biocon) (PSF)	70	Abs
	Hypocreales_fam_Incertae_sedis (3, 5)	*Acremonium* (BioRm)	75	100
		Sarocladium (PP)	25	Abs
	Nectriaceae (10, 24)	*Fusarium* (BioFrt) (PP)	66	60
		Gibberella (PGPF)	20	40
	Periconiaceae (1, 5)	*Periconia*	100	100
	Thyridariaceae (1, 5)	*Roussoella* (Decomp)	100	100
	Trichomeriaceae (1, 5)	*Knufia* (PGPF)	100	100
	Apiosporaceae (1, Abs)	*Arthrinium*(Decomp)	100	Abs
	Bionectriaceae (2, Abs)	*Bionectria* (PP) (PhtHr)	12	Abs
		Clonostacys (Biocon)	38	Abs
		Nectriopsis	50	Abs

	Chaetosphaeriaceae (2, Abs)	*Chloridium*	20	Abs
		Dictyochaeta *(Decomp)*	80	Abs
	Coniochaetaceae (3, Abs)	*Coniochaeta* *(Decomp)*	50	Abs
		Lecythophora *(HgDeTox)*	50	Abs
	Cordycipitaceae (1, Abs)	*Cordyceps (BioFert)*	100	Abs
	Cyphellophoraceae (2, Abs)	*Cyphellophora* *(N-Ut)*	100	Abs
	Dictyosporiaceae (1, Abs)	*Neodendryphiella* *(PP)*	33	Abs
		Pseudocoleophoma	33	Abs
	Didymosphaeriaceae (4, Abs)	*Pseudopithomyces* *(DrtTol)*	57	Abs
		Paraconiothyrium *(MnOx)*	36	Abs
		Bimuria (LigCDgrd)	7	Abs
	Lasiosphaeriaceae (2, Abs)	*Arnium*	25	Abs
		Echria	25	Abs
	Lentitheciaceae (1, Abs)	*Keissleriella (Biocon)*	33	Abs
		Poaceascoma *(LigCDgrd)*	33	Abs
	Lindgomycetaceae (2, Abs)	*Chlohesyomyces*	80	Abs
		Hongkongmyces	20	Abs
	Myrmecridiaceae (1, Abs)	*Mymecridium (PP)*	100	Abs
	Onygenaceae (1, Abs)	*Polytolypa (Biodg)*	100	Abs
	Ophiocordycipitaceae (2, Abs)	*Purpureocillium* *(PGPF) (PSF)*	80	Abs
	Phaeomoniellaceae (2, Abs)	*Pseudophaeomonie lla*	100	Abs
	Phaeosphaeriaceae (4, Abs)	*Didymocyrtis*	15	Abs
		Parastagosphora *(PP)*	8	Abs
		Phaeosphaeria *(Biodg)*	16	Abs
		Phaeosphaeriopsis *(PhtHr)*	8	Abs
		Setophoma (PP)	16	Abs
		Wojnowiciella	8	Abs
	Plectosphaerellaceae (1, Abs)	*Lectera*	100	Abs
	Pleosporaceae (1, Abs)	*Alternaria (PP)*	30	Abs
		Bipolaris (PP)	13	Abs
		Curvularia (BioFert) *(DrtTol)*	100	Abs

		Exserohilum (Biocon)	4	Abs
	Pleosporales_fam_Incertae_sedis (1, Abs)	*Pseudorobillarda* (Biodg)	100	Abs
	Rhytismatales_fam_Incertae_sedis (1, Abs)	*Karstenia*	100	Abs
	Sclerotiniaceae (1, Abs)	*Clarireedia* (PP)	100	Abs
	Sporocadaceae (6, Abs)	*Discosia* (PSF)	100	Abs
	Sporormiaceae (1, Abs)	*Westerdykella* (Biodg)	100	Abs
	Stachybotryaceae (1, Abs)	*Stachybotrys* (Biodg)	100	Abs
	Stictidaceae (1, Abs)	*Neofitzroyomyces*	100	Abs
	Sympoventuriaceae (1, Abs)	*Scolecobasidium* (BioRm)	100	Abs
	Tetraplosphaeriaceae (1, Abs)	*Tetraplosphaeria*	100	Abs
	Torulaceae (1, Abs)	*Torula* (Biodg) (PSF) (PGPF)	100	Abs
	Trichocomaceae (1, Abs)	*Talaromyces* (PSF)	75	Abs
		Thermomyces (ChitinDg) (PSF)	25	Abs
	Trichosphaeriaceae (1, Abs)	*Niagosphora*	100	Abs
	Xylariaceae (1, Abs)	*Annulohpoxylon* (Biocon)	100	Abs
Basidiomycota	Agaricaceae (3, 2)	*Chlorophyllum* (BioFert)	Abs	100
		Lepiota (Biodg-sap)	50	Abs
	Cortinariaceae (8, 85)	*Cortinarius* (N-Ut)	100	100
	Hydnodontaceae (12, 6)	*Disciseda* (Sltst)	Abs	100
		Trechispora	100	Abs
	Lycoperdaceae (11, 6)	*Arachnion*	38	Abs
		Holocotylon	25	Abs
		Lycoperdon (Biodg-sap)	25	Abs
	Bolbitiaceae (1, Abs)	*Conocybe* (PP)	100	Abs
	Bondarzewiaceae (1, Abs)	*Heterobasidion* (Biodg) (PP)	100	Abs
	Bulleribasidiaceae (1, Abs)	*Hannaella*	100	Abs
	Corticiaceae (4, Abs)	*Limonomyces* (PP)	100	Abs
	Cyphellaceae (3, Abs)	*Henningsomyces* (LigDgrd)	100	Abs
	Entolomataceae (3, Abs)	*Clitopilus* (ZnSF) (FeSF)	100	Abs
		Entoloma	100	Abs

	Erythrobasidiaceae (14, Abs)	*Bannoa* *(Biodg)* *(FermAg)*	100	Abs
	Malasseziaceae (3, Abs)	*Malassezia* *(Path)*	100	Abs
	Nidulariaceae (11, Abs)	*Cyathus* *(Biodg-sap)*	100	Abs
	Piskurozymaceae (1, Abs)	*Solicoccozyma* *(PsychT)*	100	Abs
	Psathyrellaceae (3, Abs)	*Coprinellus* *(HMAccm)*	100	Abs
	Russulaceae (3, Abs)	*Russula* *(MHB-F)*	100	Abs
	Schizoporaceae (5, Abs)	*Oxyporus* *(antVOCs)*	100	Abs
	Sporidiobolaceae (3, Abs)	*Rhodotorula* *(Biodg)* *(PSF) (PGPF)*	100	Abs
	Strophariaceae (3, Abs)	*Stropharia*	100	Abs
	Ustilaginaceae (5, Abs)	*Anthracocystis*	25	Abs
		Ustilago *(PP) (FeRd)* *(Antbio)*	50	Abs
Chytridiomycota	Rhizophlyctidaceae (27, Abs)	*Rhizophlyctis* *(CmCbDm)*	100	Abs
	Spizellomycetaceae (37, Abs)	*Spizellomyces* *(PP)*	75	Abs
Mortierellomycota	Mortierellaceae (87, 100)	*Mortierella* *(BioFert)*	100	100
	Rhizopodaceae (13, Abs)	*Rhizopus* *(HydDeg)(PSF)*	100	Abs
Kickxellomycota	Kickxellaceae (100, Abs)	*Ramicandelaber*	100	Abs
Glomeromycota	Glomeraceae (100, Abs)	*Oehlia* *(AMF)(DrtTol)*	67	Abs
	Paraglomeraceae (100, Abs)	*Paraglomus* *(AMF)* *(DrtTol)*	100	Abs

Abbreviations for bacterial functions within EI and EF rhizosphere soil: AMF; Arbuscular Mycorrhiza Fungi, Biocon; Bio-controller, BioFert; Bio-fertilizers, PSF; Phosphorus Solubilizing Fungi, BioRm; Bio-remediation, BioDg; Biodegrader, LigCDgrd; Ligno-cellulose Degradation, Decomp; Decomposer, Sidphr; Siderophore producers, N-Ut; Nitrogen Utilizer, PP; Plant Pathogen, MnOx; Manganese Oxidizers, Metblt; Secondary Metabolizers , PGPF; Plant Growth Promoting Fungi, PhtHr; Phytohormone Producer, HgDeTox; Mercury De-toxifying, DrtTol; Draought Tolerance, ChitiDg; Chitin Degrader, Sltst; Salt Stress, Biodg-sap; Bio degrader – saprophyte, ZnSF; Zinc Solubilizing Fungi, FeSF; Iron Solubilizing Fungi, FermAg; Fermentating Agent, Path; Pathogen, PsychT; Cold Tolerance , FeRd; Iron Reducing Fungi, Antbio; Anti-biosis, CmCbDg; Complex-Carbohydrate degrader, HydDeg; Hydrocarbon Degradation; and, Abs = Absent.
